# EGFR tyrosine kinase inhibitors promote pro-caspase-8 dimerization that sensitizes cancer cells to DNA-damaging therapy

**DOI:** 10.18632/oncotarget.3959

**Published:** 2015-04-29

**Authors:** Yun-Tian Li, Xiao-Jun Qian, Yan Yu, Zhen-Hua Li, Rui-Yan Wu, Jiao Ji, Lin Jiao, Xuan Li, Peng-Fei Kong, Wen-Dan Chen, Gong-Kan Feng, Rong Deng, Xiao-Feng Zhu

**Affiliations:** ^1^ State Key Laboratory of Oncology in South China, Collaborative Innovation Center for Cancer Medicine, Cancer Center, Sun Yat-sen University, Guangzhou, China; ^2^ Department of Oncology, Anhui Provincial Hospital, Affiliated to Anhui Medical University, Hefei, China; ^3^ The School of Medicine, Jinan University, Guangzhou, China

**Keywords:** EGFR inhibitors, doxorubicin, breast cancer, caspase-8, sequential application

## Abstract

The combination of time and order-dependent chemotherapeutic strategies has demonstrated enhanced efficacy in killing cancer cells while minimizing adverse effects. However, the precise mechanism remains elusive. Our results showed that pre-treatment of MCF-7 and MDA-MB-468 cells with epidermal growth factor receptor (EGFR) tyrosine kinase inhibitor erlotinib or lapatinib significantly enhanced the cytotoxic effects of DNA-damaging agents compared to coadministration of the EGFR inhibitor and DNA-damaging agent. Sequential application of erlotinib and doxorubicin increased activated caspase-8 by promoting pro-caspase-8 homodimerization and autocatalytical cleavage, whereas coadministration did not. We found that EGFR inhibitors promoted pro-caspase-8 homodimerization by inhibiting ERK pathway signaling, while doxorubicin promoted it. Our data highlight that ERK has the potential to inhibit the formation of pro-caspase-8 homodimers by phosphorylating pro-caspase-8 at S387. In conclusion, the pretreatment of EGFR tyrosine kinase inhibitors promote pro-caspase-8 dimerization that sensitizes cancer cells to DNA-damaging agents. Our findings provide rationale for novel strategies for the implementation of combined targeted and cytotoxic chemotherapy within a new framework of time and order-dependent therapy.

## INTRODUCTION

Over the past decades, several important, physiologic mechanisms of cell death have been described: (1) Apoptosis, a mechanism of programmed cellular death, involves two major pathways: the ‘extrinsic’ and ‘intrinsic’ pathway [1, 2]; (2) Autophagy, is known as a non-apoptotic model of cell suicide but the details regarding its underlying process remain controversial [3, 4]; (3) Necroptosis, like apoptosis and autophagy, is controlled by a regulated program but characterized microscopically by a necrotic phenotype [5, 6]. Activation of any of the above pathways is a potentially catastrophic event for the cell and remains one of the mechanisms by which a malignant cell can kill itself in the presence of a drug [7]. A better understanding of the mechanisms by which anti-cancer drugs exert such effects is essential to improving the efficacy of combination therapies and limiting the likelihood of resistance development. Doxorubicin (DOX) is a major anthracycline chemotherapeutic agent used in the treatment of breast cancer despite dose-limiting adverse effects, such as cardiotoxicity, and the potential for facilitating the development of multidrug resistance [8, 9]. To address these limitations and improve its efficacy, DOX is often supplemented by combination with other chemotherapeutic agents [10]. However, this genotoxic combination induces the intrinsic apoptosis pathway through DNA damage additionally [11, 12]. Thus, the combined use of chemotherapies with similar mechanisms of action has limited efficacy and can potentially facilitate the development of drug resistance.

Novel targeted therapies have shown considerable clinical efficacy with improvements in overall survival across a spectrum of human cancers [13-15]. The potential efficacy of a novel, combined therapeutic strategy utilizing tyrosine kinase inhibitors (TKIs) alongside cytotoxic chemotherapy has previously been explored in the treatment of breast cancer. However, EGFR inhibition in combination with genotoxic agents such as cisplatin have resulted in less than a 10% survival benefit [16]. Moreover, the addition of EGFR inhibitor cetuximab to carboplatin did not improve outcomes in a randomized phase II trial in triple negative breast cancer (TNBC) patients [17]. While these results are far from encouraging, experimental data indicate that time-staggered EGFR inhibition, as opposed to simultaneous co-administration, can dramatically sensitize a subset of triple-negative breast cancer cells to genotoxic drugs [18]. The same phenomenon has also been demonstrated in non-small cell lung carcinoma (NSCLC). In four randomized phase III trials [19, 20], while concurrent administration of erlotinib or gefitinib with standard platinum-doublet chemotherapy did not improve survival compared with chemotherapy alone, the sequential, staggered scheduling of erlotinib followed by cytotoxic chemotherapy led to a significant improvement in progression-free survival (PFS) in patients with advanced NSCLC, in the multicenter, randomized phase II First-Line Asian Sequential Tarceva and Chemotherapy Trial (FAST-ACT) [21]. Pre-clinical evidence indicates a potential antagonism that exists between the constituents of such combination therapies when they are administered simultaneously [22]. On the other hand, the molecular mechanism underlying the efficacy of sequential co-administration has not been elucidated.

It has been shown that erlotinib-dependent caspase-8 activation occurs following DNA damage, which activates the intrinsic apoptotic pathway, but the underlying molecular mechanism remains elusive [18]. Caspase-8 activation through dimerization is known to recruit oligomeric activation platforms that assemble subsequent to activation of the extrinsic pathway [23-25]. It is also well-established that caspase-8 phosphorylation induces the formation of a stable, inactive cytosolic dimer, and this hypothesis has been proven through Lyn induced pro-caspase-8 phosphorylation and dimerization [26]. The aim of our work was to identify cytosolic proteins affected by EGFR inhibition that promote caspase-8 activation in a breast cancer model. We found that procaspase-8 activation was induced by EGFR inhibitors, with subsequent activation of the downstream caspase-dependent pathways, including both the extrinsic and the intrinsic apoptotic cascades. Our findings demonstrate a potential mechanism underlying the efficacy of sequential scheduling of combined TKIs and genotoxic chemotherapy administration.

## RESULTS

### Sequentially application of EGFR inhibitor followed by doxorubicin mediated cell death

**Figure 1:**
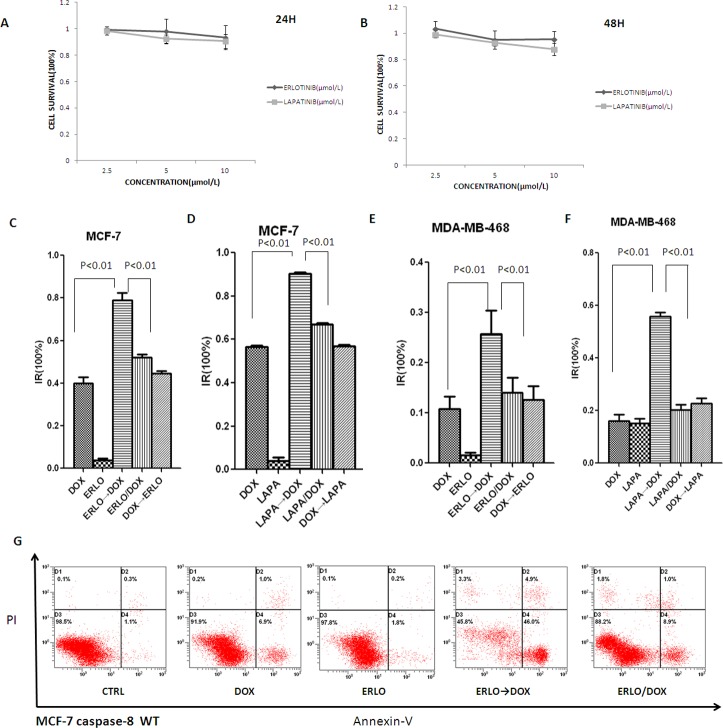
Sequential scheduling of EGFR inhibitors with doxorubicin in breast cancer cells **A, B.** Inhibitory effects of erlotinib and lapatinib on MCF-7 cell proliferation. Cell growth was assessed using the MTT assay after treatment with erlotinib or lapatinib for 24hr or 48hr. **C**–**F.** Inhibitory effects of sequential combination of EGFR inhibitors with doxorubicin on MCF-7 and MDA-MB-468 cells proliferation. ERLO→DOX or LAPA→DOX refer to erlotinib or lapatinib given 24h before doxorubicin; DOX→ERLO or DOX→LAPA refer to doxorubicin given 4h before erlotinib or lapatinib; ERLO/DOX or LAPA/DOX refers to erlotinib or lapatinib and doxorubicin added at the same time. For each combination, cell growth was assessed using the MTT assay made 12 hr after the addition of doxorubicin. IR: inhibitory rate. The concentrations of erlotinib, lapatinib and doxorubicin were used at 10μM, unless stated otherwise. **G.** Apoptosis induced by sequential combination of EGFR inhibitors with doxorubicin on MCF-7. ERLO→DOX refer to erlotinib given 24h before doxorubicin; ERLO/DOX refers to erlotinib and doxorubicin added at the same time. For each combination, apoptosis rate was analyzed by the flow cytometry assay.

The growth inhibition of the EGFR inhibitors erlotinib and lapatinib in the breast cancer MCF-7 cells was examined. Surprisingly, both erlotinib and lapatinib did not yield an apparent effect on MCF-7 cell viability, even at 10 μM concentration and 48 h incubation (Figure 1A and 1B). This negligible inhibition indicates that MCF-7 cells exhibit strong resistance to EGFR inhibition. To improve the growth inhibition of MCF-7 cells, the time-staggered administration of EGFR inhibitors was conducted followed by DNA intercalating agent doxorubicin, which has been reported to dramatically inhibit a subset of triple-negative breast cancer cells. We found that 10μM doxorubicin could reduce cell proliferation by 30-50% (Figure 1C and 1D). Simultaneous co-administration showed no difference compared to doxorubicin alone. Interestingly, however, combinations in which EGFR inhibitors were administered 24 h prior to doxorubicin demonstrated a markedly enhanced inhibition of proliferation, with an inhibitory rate of 70-90%. When the order of drug presentation was reversed— doxorubicin given before erlotinib – cell killing was not enhanced relative to treatment with doxorubicin alone (Figure 1C and 1D). We next studied cell death responses to the combination of erlotinib and doxorubicin by AnnexinV-PI staining (Figure 1G). Consistent with the MTT assays, time and order-dependent combination of erlotinib and doxorubicin led to a markedly enchanced apoptotic rate. Our results are consistent with a prior study by Lee et al. [18].

Although EGFR is overexpressed in MDA-MB-468 cells, they were also insensitive to EGFR inhibition. To verify and validate the efficacy of the order and timing of drug administration in this model, the potential for synergistic killing was also explored following time-staggered pre-treatment with either erlotinib or lapatinib in combination with doxorubicin. As expected, pre-treatment with EGFR inhibitors could also sensitize the MDA-MB-468 cells to doxorubicin (Figure 1E and 1F).

### Sensitization to doxorubicin-induced cell death by pre-treatment with erlotinib is mediated via caspase-8

**Figure 2:**
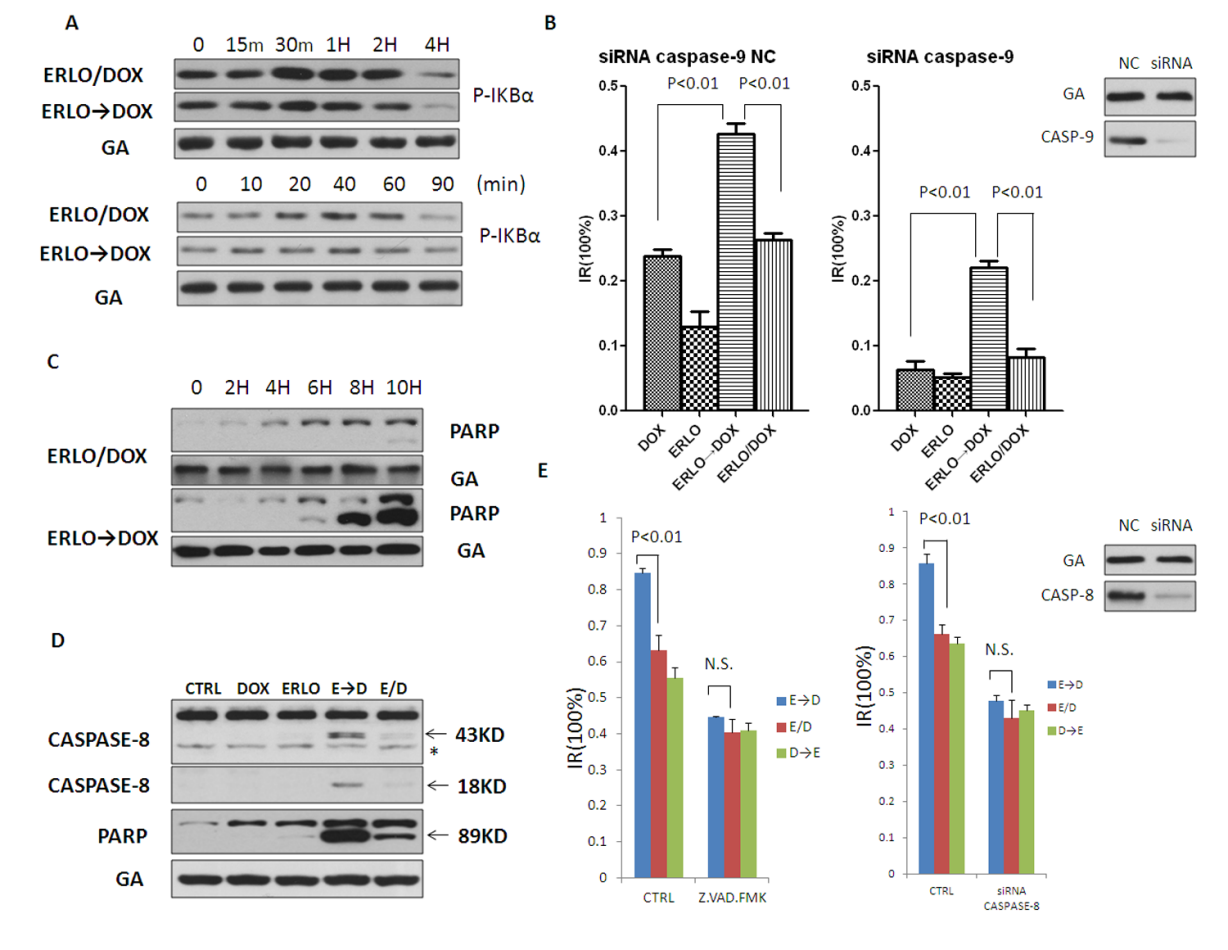
Sensitization for doxorubicin-induced cell death by pretreatment with erlotinib is mediated via caspase-8 **A.** Immunoblot analysis of phospho-IKBα in the ERLO→DOX and ERLO/DOX treated cells. MCF-7 cell extracts were collected at indicated times after the addition of doxorubicin. **B.** MTT assay assessing the sensitivity of DOX, ERLO→DOX and ERLO/DOX after knockdown of caspase-9 by siRNA. **C.** Immunoblot analysis of cleaved PARP in the ERLO→DOX and ERLO/DOX treated cells. MCF-7 cell extracts were collected at indicated times after the addition of doxorubicin. **D.** Immunoblot analysis of cleaved caspase-8 and cleaved PARP in the DOX, ERLO, ERLO→DOX and ERLO/DOX treated cells. MCF-7 cell extracts were collected 6 h after the addition of doxorubicin. Asterisk indicates nonspecific binding of Abs. **E.** MTT assay assessing the sensitivity of ERLO→DOX, DOX→ERLO and ERLO/DOX after treated with caspase-8 inhibitor Z.VAD.FMK (20μM) or knockdown of caspase-8 by siRNA. N.S.: no significance.

We next sought to identify the molecular basis for the observed difference in therapeutic efficacy between simultaneous co-administration versus sequential dosing. Activation of the NF-κB family of transcription factors is known to mediate cellular responses to DNA-damaging therapies; however, there was no apparent difference in the activation of the NF-κB pathway between these two groups (Figure 2A). As DNA damaging agents commonly induce apoptosis through activation of caspase-9, key mediator of the intrinsic apoptosis pathway, we investigated the role of caspase-9 in the sequential, combined administration of erlotinib and doxorubicin. Interestingly, although silencing of caspase-9 by siRNA reduced the inhibitory rate of both simultaneous and sequential co-administration, the staggered, sequential treatment strategy was still effective (Figure 2B). Then the extrinsic apoptotic pathways were analyzed. The results of Western blotting revealed that pre-treatment with erlotinib followed by doxorubicin markedly increased apoptosis in MCF-7 cells compared with simultaneous co-administration, as evidenced by higher levels in cleaved PARP after sequential treatment (Figure 2C). Furthermore, levels of the activated form of caspase-8, intermediate fragments p43/p41 and p18, were also increased in sequential dosing (Figure 2D). Based on these findings, the role of the initiator caspase of the extrinsic apoptotic pathways, caspase-8, was further investigated. Treatment of MCF-7 cells with a pan-caspase inhibitor, Z.VAD-FMK, significantly suppressed doxorubicin-induced cell death and attenuated the difference between simultaneous co-administration and sequential dosing (Figure 2E left). To confirm the involvement of caspase-8 in erlotinib-mediated chemosensitization to doxorubicin, MCF-7 cells with silencing of caspase-8 by siRNA was preformed. Consistent with the result of Z.VAD-FMK administration, silencing of caspase-8 also significantly suppressed doxorubicin-induced cell death and attenuated the difference between co-administration and sequential dosing (Figure 2E right). These results indicated that pre-treatment with erlotinib sensitized MCF-7 cells to doxorubicin by inducing the caspase-8 mediated extrinsic apoptotic pathway.

### Erlotinib promotes pro-caspase-8 homodimerization

**Figure 3:**
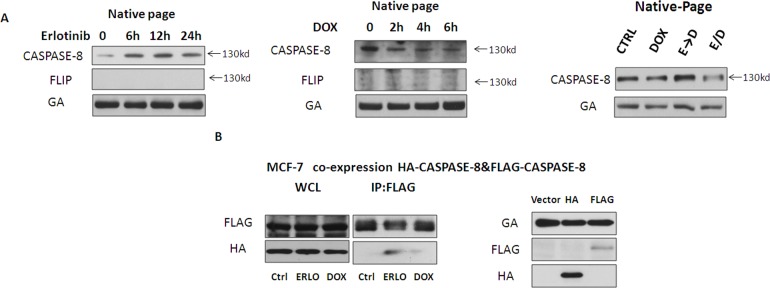
Erlotinib promotes pro-caspase-8 homodimerization **A.** Cell lysates were analyzed by native-page Western blot to detect dimerization of pro-caspase-8 in MCF-7 cells. MCF-7 cells were treated with erlotinib, doxorubicin, ERLO→DOX or ERLO/DOX. For ERLO→DOX and ERLO/DOX, MCF-7 cell extracts were collected 6 h after the addition of doxorubicin. **B.** Coimmunoprecipitation assay to test for interactions between FLAG-caspase-8 and HA-caspase-8 in MCF-7 cells by transfected Vector, HA-caspase-8 and FLAG-caspase-8.

It is well known that activation of pro-caspase-8 results from its homodimerization. We asked whether erlotinib may have an effect on the dimerization of pro-caspase-8 in MCF-7 cells. To investigate this issue, the native-page was performed, and the band around the 130KD mark was regarded as a dimer of caspase-8, which has been previously reported and validated. MCF-7 cells were treated with either erlotinib or doxorubicin respectively at indicated times. Interestingly, we found that erlotinib increased levels of this caspase-8 dimer while doxorubicin reduced it (Figure 3A). To demonstrate that this was indeed a homodimer, a homolog of caspase-8, FLIP, which lacks a functional caspase domain but can form a heterodimer with caspase-8, was detected simultaneously. Indeed, FLIP was not detected at the same position around 130KD, confirming that this dimer was the homodimer of caspase-8. Furthermore, we constitutively expressed FLAG-caspase-8 and HA-caspase-8 in MCF-7 cells. Co-immunoprecipitation revealed that erlotinib increased the combination of Flag-caspase-8 and HA-caspase-8, which was consistent with our native-page result (Figure 3B). Taken together, these data indicate that pre-treatment with erlotinib sensitized MCF-7 cells to doxorubicin by promoting pro-caspase-8 homodimerization and activity.

### Erlotinib induces pro-caspase-8 homodimerization by inhibiting pERK 1/2

**Figure 4:**
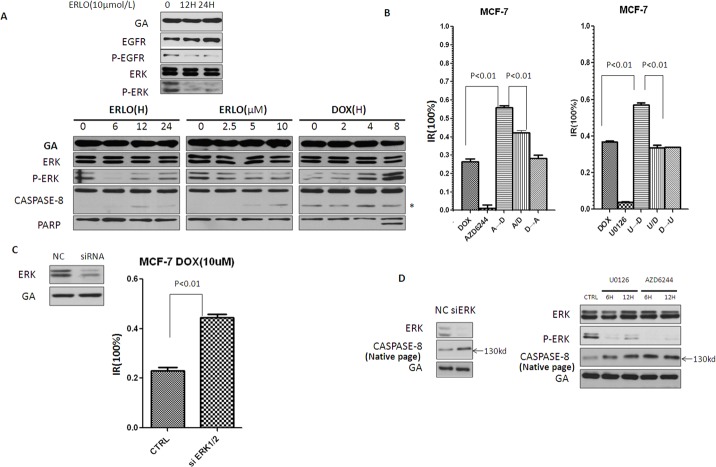
Erlotinib induces pro-caspase-8 homodimerization by inhibiting P-ERK **A.** Immunoblot analysis of the level of P-ERK in MCF-7 cells treated with erlotinib and doxorubicin. Asterisk indicates nonspecific binding of Abs. **B.** MTT assay assessing sequential combination proliferation inhibitory of MEK inhibitors (AZD6244 and U0126) and doxorubicin in MCF-7 cells. MTT assay was made 12 hr after the addition of doxorubicin. AZD6244 was treated at 5μM, U0126 was treated at 10μM, unless stated otherwise. **C.** Immunoblots of knock-down of in cells by siRNA. MTT assay assessing doxorubicin sensitivity of MCF-7 cells after ERK knockdown. **D.** MCF-7 cells were treated with MEK inhibitors and siRNA, pro-caspase8 homodimer was detected by Native-page Western blot analysis.

Dimerization plays a crucial role in the activity of many kinases, including Raf, CDK2 (cyclin-dependent kinase 2) and EGFR (epidermal growth factor receptor) [27]. However, dimerized conformational states are often stabilized by the appropriate phosphorylation of the appropriate residues. When pro-caspase-8 homodimerization and activity was investigated, Lyn was demonstrated to phosphorylate pro-caspase-8 at Tyr380, thereby inducing the formation of an inactive pro-caspase-8 homodimer, while the mechanism inducing the formation of an active pro-caspase-8 homodimer was unknown. Previous reports have suggested that caspase-8 can be phosphorylated at its S387 residue and is a substrate of P-ERK [28]. Since P-ERK is a the major downstream effector of the EGFR pathway, which could also be downregulated by erlotinib-mediated EGFR inhibition, we asked whether erlotinib could induce pro-caspase-8 homodimerization by attenuating pERK 1/2 mediated phosphorylation of pro-caspase-8 at S387. To investigate this possibility, P-ERK was analyzed in MCF-7 cells treated with either erlotinib or doxorubicin. As expected, without impacting the level of ERK, erlotinib downregulated P-ERK expression, while doxorubicin upregulated its expression (Figure 4A). Based on these findings, we hypothesized that ERK represents a key molecule in the phenomenon of sequential dosing. Thus, we replaced erlotinib with MEK inhibitors, AZD6244 and U0126, which have been reported to significantly reduce P-ERK levels. We found that ERK inhibition was sufficient to mimic the effects of erlotinib in sequential applications of anti-cancer drugs, as pre-treatment with AZD6244 or U0126 could also sensitize MCF-7 cells to doxorubicin treatment (Figure 4B). Furthermore, compared with negative controls, ERK knockdown by siRNA could also sensitize MCF-7 cells to doxorubicin treatment (Figure 4C). These results provide further, adjunctive evidence that pre-treatment with erlotinib sensitized MCF-7 cells to doxorubicin by inhibiting downstream activation of P-ERK.

To demonstrate that P-ERK reduces pro-caspase-8 homodimer, MCF-7 cells were treated with MEK inhibitors and siRNA knockdown and pro-caspase-8 homodimer levels were measured. Our data indicated that either MEK inhibition or siRNA knockdown could increase levels of this dimer (Figure 4D).

### Erlotinib induces pro-caspase-8 homodimerization by attenuating the phosphorylation of pro-caspase-8 at S387 mediated by pERK 1/2

**Figure 5:**
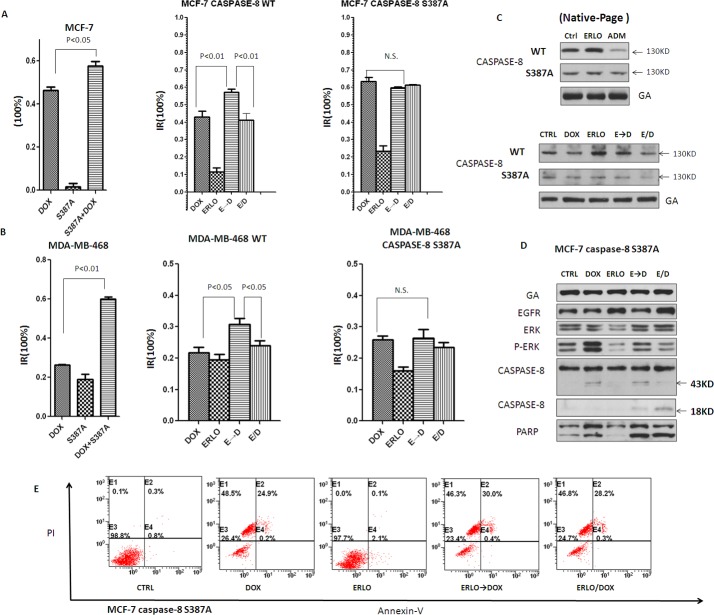
Erlotinib induces pro-caspase-8 homodimerization by attenuating the phosphorylation of pro-caspase-8 at S387 mediated by pERK 1/2 **A, B.** MTT assay assessing the proliferation inhibitory in MCF-7 and MDA-MB-468 cells transfeted with S387A mutant caspase-8 or WT treated with DOX, ERLO, ERLO→DOX and ERLO/DOX. MTT assay was made 12 h after the addition of doxorubicin. **C.** Native-page Western blot analysis of caspase-8 dimerization in the DOX, ERLO, ERLO→DOX and ERLO/DOX treated cells with or without S387A mutant pro-caspase-8 transfected. Cell lysates were collected 6h after the addition of doxorubicin. **D.** Immunoblot analysis of indicated proteins in the DOX, ERLO, ERLO→DOX and ERLO/DOX treated cells with S387A mutant pro-caspase-8 transfection. Cell lysates were collected 6h after the addition of doxorubicin. **E.** Apoptosis analysing by Annexin V-FITC/PI staining in pro-caspase-8 S387A mutant MCF-7 cells treated with erlotinib and doxorubicin or sequential application.

To examine whether phosphorylation of Ser387 by P-ERK is involved in erlotinib induced procaspase-8 homodimerization, this site was replaced with a mutant, non-phosphorylatable amino acid (S387A). The S387A mutant significantly enhanced doxorubicin-induced cell death in MCF-7 cells and MDA-MB-468 cells (Figure 5A and 5B). Moreover, the non-phosphorylatable (S387A) caspase-8 attenuated the synergistic effects of the sequential application of erlotinib and doxorubicin compared to co-administration, suggesting that caspase-8 S387 plays a key role in the synergistic effects of sequential administration of erlotinib and doxorubicin (Figure 5A and 5B). These experiments allowed us to conclude that P-ERK impaired caspase-8 activity in this system.

Next, we examined whether phosphorylation at the S387 residue by P-ERK modulates caspase-8 dimerization. The native-page showed that MCF-7 cells with caspase-8 S387A did not increase or even reduced dimerization with erlotinib or doxocubicin compared to the empty vector control (Figure 5C). Further experiment showed that the expression of caspase-8 S387A in MCF-7 cells eliminated the difference of pro-caspase-8 autocatalytic activation with doxorubicin alone, simultaneous co-administration of erlotinib and doxorubin, as well as their sequential administration. In addition, apoptosis was activated equally among these three different treatment groups, as cleaved PARP showed no apparent difference (Figure 5D). Apoptosis analysis by Annexin V-FITC/PI staining showed that caspase-8 S387A in MCF-7 cells attenuated the enhanced apoptotic effect facilitated by the sequential application of erlotinib and doxorubicin (Figure 5E), which was consistent with MTT assay and Western blot analysis.

In general, these data indicate that phosphorylation of procaspase-8 at S387 mediated by pERK 1/2 impairs erlotinib-induced procaspase-8 homodimerization as well as caspase-8 activation.

## DISCUSSION

DNA damage [29], such as treatment with doxorubicin, leads to p53-mediated induction of Noxa and Puma and the subsequent induction of the intrinsic apoptosis pathway, which is mediated by caspase-9 [30]. In this study, we found that pre-treatment with erlotinib followed by doxorubicin could lead to activation of the extrinsic apoptotic pathway, which is mediated by caspase-8, while the simultaneous co-administration of these two distinct classes of anti-cancer agents showed no induction of extrinsic apoptosis. We hypothesize that the synergistic therapeutic effect of sequential administration of EGFR inhibitor followed by doxorubicin was related to the co-induction of the intrinsic and extrinsic apoptotic pathways.

The importance of the MAPK pathway [31] in treatment response and chemoresistance has been recognized [32]. The canonical MAPK signaling cascade, depending on the extracellular signal-regulated kinases (ERK), can provide a protective effect, thereby limiting DNA damage [33]. P-ERK is known to protect cancer cells from undergoing death receptor-mediated apoptosis by phosphorylating pro-caspase-8 [28]. However, its underlying molecular mechanism has not been previously elucidated.

In this study, we provide evidence for a mechanism underlying the synergy between pre-treatment with EGFR inhibitors followed by DNA-damaging chemotherapy application in breast cancer cell lines. Upon DNA damage, cancer cells undergo defensive biological behavior tactics that promote cell survival and facilitate DNA repair, for instance, by activating several known survival pathways. We found that treatment with doxorubicin resulted in P-ERK activation and ultimately led to chemoresistance in breast cancer cells. Activated ERK inhibited caspase-8-induced apoptosis by preventing pro-caspase-8 homodimerization. Since EGFR inhibitors can reduce P-ERK levels, DNA-damaging agents in simultaneous combination with EGFR inhibitors should provide a synergistic effect. However, the co-administration of these two classes of anti-cancer agents shows no enhanced efficacy over DNA-damaging agents alone. Interestingly, our data demonstrate that pre-treatment with EGFR inhibitors can sensitize breast cancer cells to DNA-damaging agents.

Our study showed that although ERK can be inhibited through simultaneous co-administration of these two kinds of anti-cancer agents, the formation of pro-caspase-8 homodimer was not increased, whereas pre-treatment with erlotinib could increase levels of this critical dimer. Our data suggest that pre-treatment with erlotinib can increase the pro-caspase-8 homodimer and thereby sensitize cancer cells to strong apoptotic stimuli, such as doxorubicin.

Ras family proteins are essential downstream components of the EGFR/Ras/MAPK signaling pathways and Ras mutations are frequently detected in several cancers [34], thereby limiting the clinical benefit of EGFR inhibitors. In these Ras mutant cell lines, sequential application of EGFR inhibitors and doxorubicin may not demonstrate a superior, synergistic effect over their concurrent co-administration. However, our experiments suggest that replacing the EGFR inhibitors with more downstream inhibition via MEK-targeted therapy may also produce the same therapeutic effect.

In the past decades, therapeutic strategies for cancer have begun to shift from the use of individual agents to combined therapeutic regimens, and it is well-established that cytotoxic chemotherapies are most effective and can produce synergistic effects when given in combination. However, these strategies are limited by the highly complex signaling networks that enable cancer cell populations to evolve towards drug-resistant clones. Thus, identification of effective combinatorial therapeutic strategies will be vital. Our study highlights one potential synergistic mechanism underlying the superior efficacy of sequential scheduling of TKIs and cytotoxic compounds compared to concurrent co-administration and provides a rationale for designing novel combination therapies.

## MATERIALS AND METHODS

### Cell culture and reagents

Human breast cancer cells (MCF-7, MDA-MB-468) were cultured in DMEM medium supplemented with 10% fetal bovine serum in a humidified atmosphere containing 5% CO2 at 37°C. Erlotinib, Lapatinib, Doxorubicin, U0126 and AZD6244 were purchased from Selleck Chemicals. Human normal IgG was obtained from Roche (Basel, Basel-Stadt, Switzerland). GAPDH antibody was obtained from Boster Biological Technology (Wuhan, China). MTT was purchased from Sigma-Aldrich (St. Louis, Missouri), and phospho-ERK, ERK antibodies were from Santa Cruz Biotechnology (Indian Gulch, California). All other antibodies were purchased from Cell Signaling Technology (Beverly, Massachusetts). The Annexin V-FITC Apoptosis Detection Kit was from BD Sciences.

### Cell viability/proliferation

Cells were seeded in 96-well-plate. Metabolic viability was determined using MTT(Sigma) assay according to the manufacturer's protocol.

### Plasmid construction and tTransfection

The human pcDNA3-CASP8 vector was from Addgene (Plasmid 11817). pcDNA3-CASP8 vector was constructed into another destination vector or add a different epitope tag (Flag or Ha) of choice. Site-directed mutagenesis of pcDNA3-CASP8 was performed using QuikChange XL (Stratagene). Plasmid transient transfection was performed using Lipofectamine 2000 according to the manufacturer's instructions (Invitrogen).

### RNA interference

siRNA-pro-Caspase-8 and siRNA CASPASE-9 was produced by GenePharma Target Sequences for siRNA:

CASPASE-8 sense (5′-3′) : GAUACUGUCUGAUCAUCAAtt,

antisense (5′-3′) : UUGAUGAUCAGACAGUAUCcc; CASPASE-9 sense (5′-3′) : GGUGCUCAGACCAGAGAUUTT, antisense (5′-3′) : AAUCUCUGGUCUGAGCACCTT; commercial siRNA-ERK 1/2 was from Cell Signaling (6560); All siRNAs were transfected using Lipofectamine^®^ RNAiMAX Transfection Reagent (life technologies) according to the manufacturer's protocol.

### Western blot analysis and immunoprecipitation

Whole cell extracts were generated by direct lysis with 1×Cell Lysis Buffer (Cell signaling technology, #9873) adding 1 mM PMSF immediately before use. Samples were boiled by addition 6×SDS sample buffer for 10 min at 100°C and resolved by SDS-PAGE. For immunoprecipitation, cells were lysed by E1A lysis buffer [250 mM NaCl, 50 mM HEPES (pH 7.5), 0.1% NP-40, 5 mM EDTA, protease inhibitor cocktail (Roche)]. Immunoprecipitation was carried out either by incubating FLAG beads at 4°C with lysate overnight or by incubate appropriate antibody with cell lysate for 2-3 hours, followed by incubating Protein-A/G beads overnight (Roche). Immunoprecipitates were washed 3 times with cold lysis buffer and eluted with SDS loading buffer by boiling for 10 min.

### Native-page western blot analysis

For Native-PAGE, cell extracts were generated by direct lysis with 1×Cell Lysis Buffer (Cell signaling technology, #9873) adding 1 mM PMSF immediately before use. Cells were disrupted on ice (20minutes) and obtained by centrifugation (13 000 × g for 15 min at 4°C), then added 6 × loading buffer. Proteins were separated in a 8% polyacrylamide gel using a tris-glycine running buffer excluding sodium dodecyl sulphate (SDS) at 4°C. After transfer to a PVDF membrane, the membrane was blocked with 5% milk in TBST containing 0.05% Tween 20 (PBST) for 2 h, it was probed with various primary antibodies overnight at 4°C, followed by incubation with HRP-conjugated secondary antibodies for at least 2 h at room temperature, then washed in TBST and visualized with enhanced chemiluminescence reagent following the manufacturer's instructions (Thermo Fisher Scientific Inc). Immunoreactivity was detected using the Amersham ECL Prime Western Blot detection reagent (GE Healthcare, Fairfield, CT, USA) according to the manufacturer's instructions.

### Annexin V-FITC/PI staining

According to the protocol of The Annexin V-FITC Apoptosis Detection Kit(BD Sciences), Cells with different treatments were resuspended in 100μl 1×Binding Buffer at a concentration of ~1×10^6^ cells/ml. Add 5μl FITC Annexin V-FITC and 5μl PI, cells were incubated at room temperature for 15 min in the dark. Add 400μl of 1×Binding Buffer to each tube. Apoptosis was analysed by flow cytometry (BD Company, USA) at the wavelength of 488nm immediately(within 1 hr).

### Statistical analysis

Data are presented as the mean ± SEM unless otherwise stated. Student's t test was used to compare two groups for statistical significance analysis.
